# Invertebrate Models in Innate Immunity and Tissue Remodeling Research

**DOI:** 10.3390/ijms23126843

**Published:** 2022-06-20

**Authors:** Claudia La Corte, Nicolò Baranzini, Annalisa Grimaldi, Maria Giovanna Parisi

**Affiliations:** 1 Marine Immunobiology Laboratory, Department of Earth and Sea Sciences, University of Palermo, 90123 Palermo, Italy; claudia.lacorte@unipa.it; 2 Department of Biotechnology and Life Science, University of Insubria, Via Dunant 3, 21100 Varese, Italy; nicolo.baranzini@uninsubria.it (N.B.); annalisa.grimaldi@uninsubria.it (A.G.)

The aim of this Special Issue is to highlight the close functional and highly conserved link between innate immunity, homeostasis maintenance, inflammation, tissue remodeling and regeneration. In the last few decades, the rules on the use of vertebrates in biological trials have become increasingly stringent and, in this context, invertebrates are used as complementary animal models, considering that they are cost-effective and easy to handle. Here, original articles and reviews on cells and mechanisms involved in immunity, in the regulation of inflammation and tissue regeneration processes, focused on invertebrate models, are gathered.

Considerable efforts have been made to shed light on phylogenetic distribution and developmental patterns of wound healing and regeneration mechanisms, as well as on their ecological value. Comparative research on regeneration and its relationship to the immune system has been a long-standing concern. Most likely, the capability to regenerate whole organs and body parts concurs with the origin of multicellularity and many invertebrate models have relevant regeneration abilities, unlike in vertebrates, in which this skill is significantly reduced.

In the paradigm of acquired and adaptive immunity, anamnestic memory responses are the central feature of the adaptive immune system that evolved in our vertebrate ancestors, together with cicatrisation following tissue injury, which is disadvantageous to full regeneration.

In the last few years, the key role of the extracellular matrix (ECM) during several physiological processes has become evident, including in regeneration scenarios. Parisi et al., 2021 [1] explored regenerative events after tentacle amputation in the anthozoan *Anemonia viridis*, focusing precisely on the rearrangement of the extracellular matrix in the mesoglea of this Mediterranean benthic species. They analysed the event from a biochemical, biometric, morphological and histological perspective, in unamputated and injured polyps. In particular, modifications in mesoglea stiffness were observed during tentacle regrowth, the production of metalloproteases probably attributable to fibroblast-like cells, differentiated from amebocytes, and which could play a role in the reshaping of the ECM and the deposit and spatial arrangement of collagen I fibrils. Indeed, it might act as a scaffold and as a guide for the reconstruction of missing parts. Moreover, the regrowth of the oral tentacles was followed for 14 days at the macroscopic level. The wound closed after 24 h from the lesion, appeared less pigmented, due to the absence of the endosymbiont of *Symbiodinium* genus, lost from the tissues after injury. The sprawling regrowth was already noticeable after 7 days and new pigmented 5 mm sized tentacles were visible after 14 days from amputation. At 14 days from cut, the repigmentation was correlated to the repopulation of the tissues by the zooxanthellae, which also appear to play an important role in elongating the regenerating tentacles. In this contest, the activity of alkaline phosphatase (ALP), as a typical biochemical marker associated with regenerative events and related to nematocyte and epithelial cell differentiation in cnidarian, was quantified. The researchers showed an increase in the ALP activity in tentacle extracts in all three experimental time points. On the contrary, the enzyme amount remained at a constant level in the body extract.

Histological analyses showed, for the first time, that in *A. viridis* tentacle regeneration, mesoglea move from a laxist network to an organised one of collagen fibril bundles. In addition, immunocalization tests showed that the visco-elastic behaviour of the mesoglea from the neosynthesis of collagen type I from hyaline amebocytes bFGFR+ act as fibroblast-like cells and are involved in collagen structure arrangement.

The close link between innate immune response and wound-induced regeneration was also investigated by Bodó et al., 2021 [2], on the earthworm invertebrate model *Eisenia andrei*. They focused on, with a new point of view, its rostral and caudal regeneration after injury. The classic steps of annelid regeneration in *E. andrei* with the instantaneous closure of the wound and the completed recovery of lost tissues in four weeks were observed. Moreover, several cellular and molecular modifications in regenerating earthworms were found. Immediately after the wound closure, proliferating cells create the blastema, which appeared clearly at 4 days from injury, characterized by distinct cell types. Proliferation occurs until two weeks in regenerating earthworms.

At the same time, apoptosis occurred during the whole regenerative event, particularly intensive during the wound healing, and a cellular breakdown was detected within four weeks of regenerating anterior and posterior blastema. Visualization of smooth muscle α (αSMA) by immunofluorescence, only in intact original segments, was strongly visible and so expressed after four weeks of regeneration in the muscle layers, both in anterior and posterior blastema. These remarks appear to rise the conserved role in evolution of αSMA, given its constant de-novo expression/activation in new segments.

Histochemical methods evidenced a decrease in pattern recognition receptors and antimicrobial peptides and acid and alkaline phosphatase showed significant alterations between intact and regenerating organisms. The regeneration event appeared clear after two weeks, with an increase in cell proliferation and a reorganization of new actin fibers. Concurrently the apoptosis present during the whole process decreased in two-week regenerating organisms and high-programmed cell death in early blastema formation and in the trans-differentiation phase.

Earthworm coelomocytes were involved in segment regeneration in combination with the production of several antimicrobial peptides (AMP), improving the reparation of damaged axons in central nervous system (CNS). However, authors evidenced how immune-response-related mRNA expression was reduced compared to intact animals, common in other earthworms and in other species during regeneration.

Moreover, Homa et al., 2021 [3], in *E. andrei*, investigated the effects of a manganese metalloenzyme arginase activity in coelomocytes, subjected to several and multiple-stressors, including immunostimulant lypopolisaccharide (LPS), hydrogen peroxide (H_2_O_2_), metals ions, parasite infection, wound-healing and five days of fasting. Normally, the enzyme is found both in the animal and vegetal kingdom and it plays a key role in ureagenesis, tissue regeneration and in macrophage polarization. An upregulation in the enzyme expression was related to cell growth and collagen synthesis, motility and detoxification from free-radical ions.

Overall, this study suggested the arginase role in the dual aspect of the post-injury process: immune response involvement and wound healing. Indeed, it was observed that in coelomocytes affected by LPS, hydrogen peroxide and ions up-regulate the arginase activity in in-vitro stimulation, as well as in infected specimens with nematodes or subjected to segment amputation. No remarks were evidenced in fasting organisms for five days; indeed, the number and the composition of coelomocytes did not change. Furthermore, the study confirmed that the coelomocytes metalloenzyme is suppressed by L-norvaline.

In this Special Issue, Mosca and co-authors [4] reported, through molecular and immunological markers, the biochemical and functional characterization of the endocannabinoid system in the bivalve *Mytilus galloprovincialis* haemocytes. The authors evidenced the effects on cell motility and the consequent activation of the respiratory burst. Anandamide (AEA) and capsaicin were demonstrated to inhibit the immunity response mediated by the haemocytes, and the selective antagonists CB_2_ and TRPV1 receptors revert their inhibitory effects. There was a clear morphological shift, from ameboid to rounded shape, in *M. galloprovincialis* haemocytes after the exposure to AEA and 2-AG. The phagocytosis tests performed exhibited the capability of AEA to control respiratory burst with a diminution in luminescence generation. The suppressive effects of AEA on haemocyte phagocytosis were observed at 10 nM and 100 nM in a dose-dependent manner, and the lowest concentration (1 nM) was inefficient. Thus, it was shown that the haemocytes of the mussel are not only able to synthesize, degrade and transport the major eCBs, but are also able to express the functional eCBs-binding CB_2_ and TRPV1, but not CB_1_, receptors and also exposed the suppressive impact on phagocytosis caused by capsaicin.

Another interesting step forward in the complex field of innate immune system was addressed by Yakovenko et al., 2021 [5], in their study, focused on the sea urchin *Paracentrotus lividus*, where the authors deepened the protein family of Transformer protein (Trf) involved in the immune response to pathogens. The Trf family is highly differentiated and shows several Trf sizes and conformations and these were individuated both in cell-free coelomic fluid and in coelomocytes. The authors characterized, through FACS, five sub-populations with PlTrf protein expression on the cellular membrane. The amount of these cells was higher if the organism was exposed to *Escherichia coli*. No differences in relative abundance were found in immune challenge with LPS or *Vibrio penaeicida*, a common sea urchin pathogen. They also demonstrated that the phagocytosis activity against E. coli is inhibited, blocking the functionality of PlTrf present on the cell surface with the anti-Trf antibodies. These results suggest the collaboration of humoral and cell-mediated response to bacterial challenge in *P. lividus*. Lastly, in spite of the differences between gene and protein sequences within this class, their properties and mechanisms of action, as pointed out by the authors, seem to be preserved.

Bergamini et al., 2021 [6] provided fascinating outcomes in their survey on a regenerative event in *Pomacea canaliculata*. They followed three months of the regeneration process for an adult cephalic tentacle, normally used in food searching, co-specific recognition and orientation. After 72 h post amputation, the authors showed the missing parts recognizable and the epithelial, the connective, the muscular and the neuronal tissues were distinguishable. They also showed that haemocytic recruitment occurs in the early stages of regeneration and, to quantify the number of cells present in the regenerated organs, applied an innovative and specific method. Particularly, they applied the protocol for computer-assisted image analysis to tissue sections stained with haematoxylin–eosin of specimens in regeneration. A significant amount of granular haemocytes in proximity to the blastema 12 h post amputation (hpa), followed by a fast visualized decrease of 24, 48 and 72 hpa.

The strength of the protocol developed by Bergamini and co-authors aimed at counting cells in the tissue is due to the absence of the use of a specific cellular marker. Therefore, it represents a valid tool for studying the involvement of immune cells in the regenerative process.

Clear results, as in each study reviewed here, are fundamental and useful for the study of regeneration. They provide new methods and important biological information that could be exploited in the medical or biotechnology fields, also exploiting the use of new animal models.

## Data Availability

Not applicable.
